# Mapping the landscape of mathematical models for antimicrobial resistance: a scoping review

**DOI:** 10.1186/s12889-026-27533-8

**Published:** 2026-07-02

**Authors:** Felipe Schardong, Claudio Jose Struchiner, Luiz Max Carvalho

**Affiliations:** School of Applied Mathematics, Getulio Vargas Foundation, Praia de Botafogo, 190, Rio de Janeiro, 22250-900 Brazil

**Keywords:** Mathematical modelling, Drug resistance, Literature review, Epidemiology

## Abstract

**Background:**

Antimicrobial resistance (AMR) is a serious global public health problem, that contributed to an estimated 4.95 million deaths in 2019 and with approximately 10 million annual deaths and up to US$100 trillion in cumulative economic losses projected by 2050. Its emergence and spread result from complex interactions between biological, ecological, and socioeconomic factors. Mathematical modelling has been recognized as a crucial tool for clarifying the dynamics of AMR emergence and transmission. However, the dominant literature is fragmented and characterized by notable methodological and contextual limitations. This scoping review aims to synthesize and analyse recent mathematical modelling studies on AMR to identify prevalent trends, methodological biases, and key research gaps.

**Methods:**

We conducted a scoping review following the PRISMA-ScR statement. We systematically searched three databases (PubMed, Web of Science, and Scopus) from 2019 – 2024 for published papers that created or used dynamic mathematical models of AMR. After removing duplicates and screening, 36 studies were considered eligible for inclusion. Data were extracted via a structured form that was divided into three categories: model type and context, model construction and correlated parameters, and model outputs and validation. In each category, the information considered most relevant for further analysis was extracted.

**Results:**

Our analysis demonstrated a predominance of deterministic models using ordinary differential equations (ODEs), which were mostly focused on bacterial pathogens such as *Pseudomonas aeruginosa*, *Escherichia coli*, and *Staphylococcus aureus*. The vast majority of models focused on the human host, with only one study adopting a One Health approach. The most commonly modelled resistance mechanisms are horizontal transfer by conjugation and mutation, and the rarely modelled mechanisms include transduction, transformation, host immunity, and spatial heterogeneity. Furthermore, only two have considered economic impact. There was apparent consistency in geographic inequality, with the vast majority of studies originating from high-income countries.

**Conclusion:**

Mathematical modelling of AMR is an active field, but is characterized marked by low methodological diversity and is limited in scope to a few contexts. Given these limitations, there is a need to develop mathematical models of AMR that are capable of capturing the complex dynamics among hosts, environments, transmission, and intervention dynamics. The use of deterministic models based on ODEs contributes significantly to advancements in the study of AMR dynamics, but future work requires the integration of stochasticity, spatial structure, and ecological interactions to more realistically represent the complexity of the real world. Furthermore, the introduction of a One Health framework and the incorporation of economic and social variables will be essential for the development of models that not only explain the observed patterns but also guide effective global strategies to mitigate the impact of AMR.

**Supplementary Information:**

The online version contains supplementary material available at https://doi.org/10.1186/s12889-026-27533-8.

## Introduction

Antimicrobial resistance (AMR) occurs when microorganisms develop mechanisms that reduce the efficacy of antimicrobial agents [1]. Recognized by the World Health Organization (WHO) as one of the 10 greatest global threats of the century and known as the ‘Silent Pandemic’, AMR compromises human and animal health; affects the environment, food, and nutritional security; and hinders economic development and social equality, requiring urgent action and effective management strategies [2]. While economic analyses from 2014 and 2016 projected that AMR could generate a cumulative impact of up to $100 trillion in global gross domestic product (GDP) by 2050 [3, 4], more recent global epidemiological forecasts estimate that, by 2050, AMR will be responsible for approximately 1.91 million deaths annually directly attributable to resistance and 8.22 million deaths associated with resistant infections [5]. Others recent studies [5, 6], using data from 2019 and trends from 1990 – 2021, have confirmed the severity of the problem, particularly in low- and middle-income countries (LMICs), with estimates predicting millions of deaths associated with AMR in the next decades. The rise of AMR reduces the efficacy of antimicrobial agents and represents a major threat to modern medicine, as it can result in common infections becoming difficult to treat and compromise procedures such as surgery, chemotherapy, and organ transplantation [7]. In addition, AMR causes enormous economic damage to public health systems and the economy in general [8], highlighting the need to translate existing knowledge into coordinated and effective containment strategies.

The inappropriate administration of antimicrobials among both humans and animals has long been termed one of the key factors that enhances the development of resistant pathogens and the rise of AMR [9, 10]. According to the study by Arepyeva et al. [11], and under the assumption of their statistical framework, higher antimicrobial consumption was associated with increased resistance, which is consistent with the expectation that stronger selective pressure favours resistance over susceptible subpopulations. This type of selection also depends on ecological conditions, the intensity of microbial competition, and the treatment strategies adopted, which can favour or delay the emergence and spread of AMR [12, 13]. The growth of AMR can also be attributed to genetic mechanisms [14], which involve persistent resistance mediated by genes located on chromosomes and plasmids, the latter being particularly advantageous for their ability to perform horizontal gene transfer (HGT), resulting in adaptive advantages in different ecological contexts [15]. The results from network-based and spatial structure models have shown that heterogeneous contact between bacteria, as well as the subdivision of the population into demes (subpopulations), can intensify the spread of resistance, enabling the survival of resistant strains even in hostile selective environments [16, 17].

Another determining factor in maintaining resistance is related to nosocomial infections. Hospital environments, with strong selective pressure and a high presence of antimicrobial agents, favour the endemicity of resistant strains, complicating epidemiological control [18, 19]. Mathematical models developed for these environments show that, although antimicrobials are effective against sensitive strains, inadequate treatment of patients carrying resistant strains can establish resistance in the hospital population [20]. Innovative strategies, such as therapeutic combinations, are increasingly being explored. The use of phages combined with antibiotics, for example, has shown good results and proven to be a promising alternative for combating AMR [21]. Similarly, adaptive treatments that explore competition for resources between resistant and susceptible strains can restore the effectiveness of traditional antimicrobial agents [13].

From a health policy perspective, it is widely accepted that reducing the risks associated with AMR depends on an integrated approach based on the One Health concept. This strategy enables convergence involving human, animal, and environmental health and has been proposed as key to guiding public policy and practices in governance [22, 23]. Consequently, effective policy formulation and resource allocation depend on mathematical models that effectively capture these multisectoral interactions and ecological feedbacks. Awareness campaigns are also very powerful tools for combating AMR and have had a positive impact on reducing inappropriate antimicrobial prescription and adjusting population behaviour regarding antimicrobial use, with the impact depending on the structure and duration of the campaign [24]. Another very effective method is drug risk management programs, which are recognized as key strategies for optimizing the use of antimicrobials, reducing selective pressure, and helping to prevent the spread of AMR [25].

In this context, mathematical modelling has been established as an essential tool for understanding the dynamics of AMR. Models may offer hypotheses regarding the spread and transmission of pathogens, the development and evolution of AMR, and the effects of interventions at various scopes. For example, compartmental models have been used to examine how antibiotic consumption and transmission dynamics influence resistance at the population level [26]. In applied settings, these models have supported the evaluation of antimicrobial stewardship strategies, including prescribing restrictions, cycling policies, and treatment optimization, by projecting their effects on resistance prevalence and healthcare-associated infections [27]. Evolutionary and within-host models have provided insights into the mechanisms of mutation, selection, and horizontal gene transfer leading to multidrug resistance [28]. Agent-based and network models, in turn, have been employed to capture heterogeneity in patient contact structures, ward-level transmission dynamics, and inter-hospital patient transfers, thereby informing infection prevention strategies and regional coordination policies across healthcare facilities [29]. This significant methodological variety provides varied insights into AMR, while simultaneously posing challenges in the comparison, integration, and transformation of the findings to applicable recommendations for decision-makers and policy-makers.

Despite significant advances in the mathematical modelling of AMR, research in this field remains concentrated on specific approaches. Most existing studies rely on deterministic models that are based on ODEs, whose mathematical simplicity and low computational cost facilitate initial analyses. However, this simplicity often limits the ability to capture stochasticity, individual heterogeneity, and spatial structure, factors essential for a realistic understanding of AMR dynamics and evolution. Moreover, the diversity of assumptions, data scarcity, and heterogeneous epidemiological contexts hinder the development of a unified perspective on AMR. In this context, there remains a lack of systematic synthesis regarding how these models are constructed, which assumptions and limitations recur across them, and how such constraints affect progress in the field. To address these gaps, this study conducts a scoping review of mathematical modelling applied to AMR, aiming to identify and characterize the types of models used and the pathogens and antimicrobial treatments modelled in the studies. By providing a structured overview, this review seeks to offer a conceptual framework to support the development of more realistic and integrative models capable of addressing critical gaps in current AMR mathematical modelling research.

### Background on mathematical models

Mathematical models of AMR provide a structured way to represent how sensitive and resistant populations emerge, change, and respond to selective pressures across biological scales, including antimicrobial action, microbial evolution, and ecological or epidemiological interactions [30]. Although these models differ in their levels of biological and mathematical complexity, the majority have a common goal: to capture the mechanisms that drive the emergence and spread of resistance [31]. We review the technical aspects of AMR mathematical modelling to help readers interpret the results of this review. In what follows, we provide a concise, structured summary of conceptual components that commonly characterize within-host formulations, since these elements recur across many of the approaches discussed in the text and in the papers included in this review.

A first essential component is the pharmacokinetic/pharmacodynamic (PK/PD) relationship that links the within-host time evolution of the drug concentration, typically denoted by the function *A(t)* (PK), with the effect of that concentration on the bacteria (PD), represented by a function *E(A)*. In practice, it *E(A)* is often modelled via Emax/Hill-type functions [32], which translate the intensity of bacteriostatic or bactericidal effects as a function of antimicrobial exposure. In their general (Hill) form, these functions are written as

1 $$\begin{aligned} E(A) = \frac{E_{\max } \, A^{n}}{A_{50}^{\,n} + A^{n}}, \end{aligned}$$

where $E_{\max }$ denotes the maximum achievable drug effect, $A_{50}$ is the concentration producing half of the maximum effect, and *n* (the Hill coefficient) determines the steepness of the concentration–effect curve. In the particular case *n=1*, this reduces to the classical Emax formulation, which assumes a standard saturating response without additional cooperativity. The PK component involves absorption, distribution, metabolism, and excretion (ADME) processes, describing what the body does to the drug and determining the concentration profile *A*(*t*) at the infection site over time. The PD component characterises what the drug does to the bug, that is, the relationship between drug exposure and its effect on pathogen survival or growth inhibition. The key distinction lies in their focus: PK tracks drug movement through the host, while PD quantifies the biological response of susceptible and resistant strains to that drug. Together, these two elements define the selective pressures imposed by treatment and shape the emergence of resistance, as the integration of both components is essential for predicting not only microbial killing but also the selective windows that drive resistance emergence during therapy. Despite their importance, many models simplify this PK/PD block because detailed pharmacokinetic and pharmacodynamic data are often unavailable [33], limiting the capacity to fully capture drug-host-pathogen interactions.

A second common component in within-host models concerns microbial dynamics, which are typically represented by equations describing the evolution of sensitive (*S*) and resistant (*R*) populations over time. These formulations combine terms for growth, competition, generation of resistance, antimicrobial-induced mortality, and, in some models, host immune action. Bacterial growth is often modelled with logistic-type terms such as

2 $$\begin{aligned} \alpha _i\,X\left( 1-\frac{S+R}{K}\right) , \qquad i,X \in \{S,R\}, \end{aligned}$$

while the treatment effect is incorporated via exposure-dependent mortality terms, for example $E_i(A)\,X$ with $i, X\in \{S,R\}$ [34, 35]. Resistance emergence typically appears as either (i) a mutation flux *S→ R* modelled by a term *ν S*, or (ii) HGT, which is frequently represented by conjugation and written as a mass-action term *λ S R* [36]. Conjugation is the direct transfer of plasmids (mobile genetic elements) from a donor to a recipient cell via a conjugative pilus [37]. Others HGT mechanisms are the transformation (the uptake of free extracellular DNA from the environment) and transduction (the transfer of genetic material mediated by viruses) [37]. These last two are included less frequently because they are more difficult to configure. Many models also incorporate fitness costs, implemented as a reduced growth rate for *R* (i.e., $\alpha _R<\alpha _S$). More general extensions may introduce multiple resistant classes ($R_1,R_2,\dots$) to represent resistance to different drugs (multidrug resistance, MDR) [38].

Mathematical models can be implemented via different formalisms. Deterministic models, often expressed through systems of ordinary differential equations (ODEs), describe how the mean behavior of the system evolves over time and are the most common owing to their analytical tractability and lower computational cost [35]. In contrast, stochastic models explicitly incorporate random events, such as rare mutations or sporadic HGT events, which can substantially influence the emergence of resistance, especially when pathogen populations are small [39]. Stochastic simulations, such as those generated by the Gillespie algorithm, allow the exploration of variability and uncertainty in resistance trajectories [40]. Other approaches include partial differential equation (PDE) models to capture spatial heterogeneity or agent-based models (ABMs) to simulate interactions at the individual level [41].

**Fig. 1:**
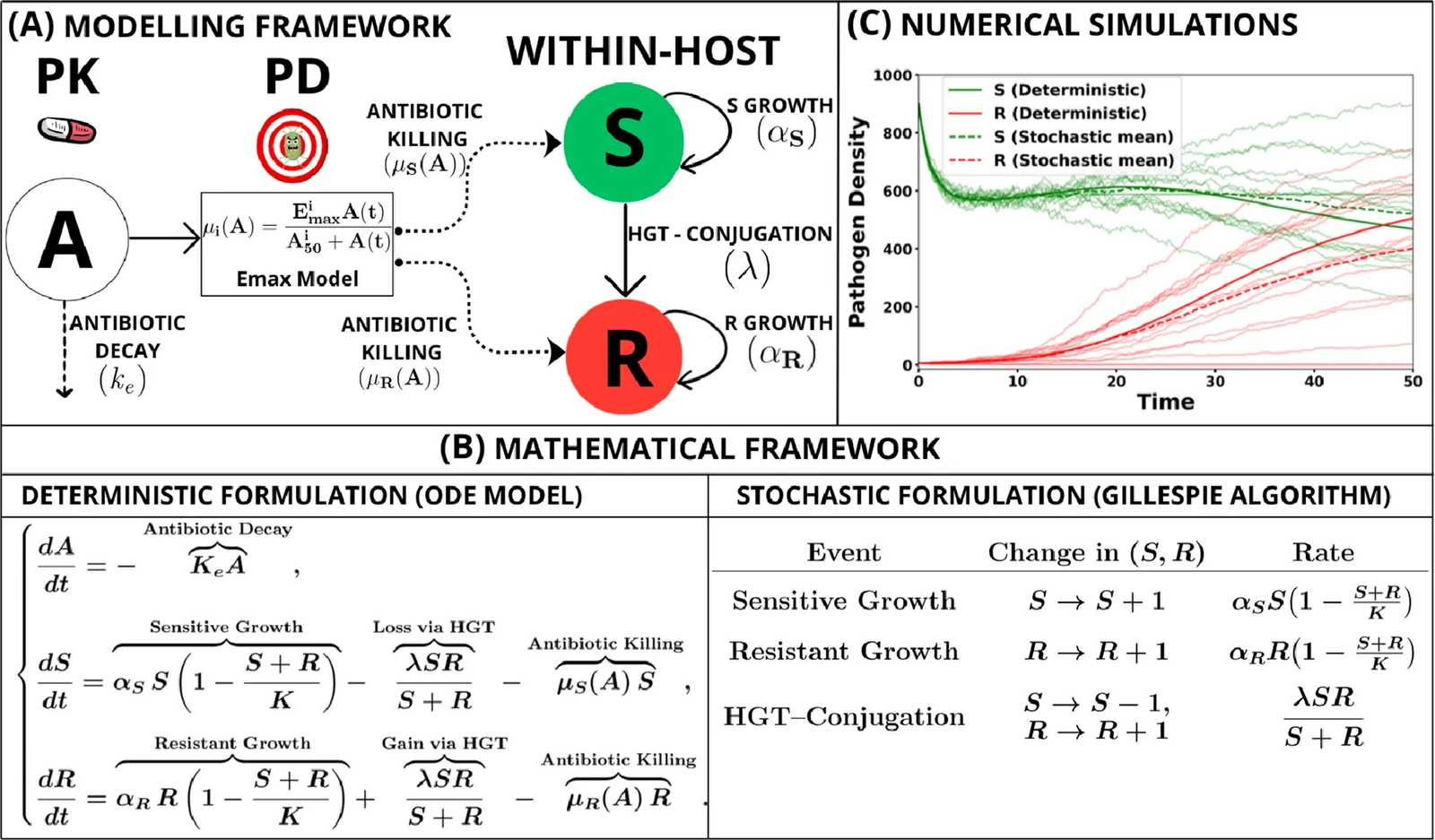
Comprehensive modelling framework for AMR, combining conceptual design, mathematical formulations, and numerical simulations. **A** Schematic representation of the full modelling framework, integrating PK/PD and within-host bacterial dynamics. The PK module governs the antibiotic concentration *A*(*t*), which feeds into the PD killing functions $\mu _i(A)$, while sensitive (*S*) and resistant (*R*) bacterial populations interact through growth, antibiotic killing, and HGT. **B** Mathematical formulations of the model. Left: deterministic ordinary differential equations describing PK, PD, bacterial growth, and HGT. Right: stochastic version based on the Gillespie algorithm, listing each event, state transition, and corresponding rate. **C** Numerical simulations comparing deterministic trajectories of sensitive and resistant populations with multiple stochastic realizations and their mean behaviour

Figure 1 summarizes these elements in a compact visual representation and shows a simplified version of an AMR model taken from Techitnutsarut & Chamchod (2021) [42], which was included in our review. Panel (A) illustrates how PK/PD dynamics and within-host pathogen dynamics are typically integrated in a single model. Panel (B) gives an example of how such systems can be formalized in deterministic (ODE) and stochastic (Gillespie) frameworks. Panel (C) shows how these formulations translate into numerical simulations, comparing mean trajectories and stochastic variability for sensitive and resistant populations. Since the goal of this exposition is purely illustrative, we adopted initial conditions and parameter values that differ from those used in the original paper; for the numerical simulations shown in panel (C) we set: $k_{e} = 0.1\,\textrm{h}^{-1}$, $\alpha _{S} = 1$, $\alpha _{R} = 0.8$, *λ = 0.01*, $E_{max}^{S} = 0.5\,\textrm{h}^{-1}$, $E_{max}^{R} = 0.2\,\textrm{h}^{-1}$, *K = 1000*, $A(0)=10\ \mu \textrm{g}/\textrm{ml}$, *S(0)=900* and *R(0)=5*.

The conceptual elements described here (PK/PD relationships, within-host dynamics, and deterministic and stochastic formulations) represent only a subset of the components that constitute mathematical models of AMR. To systematically map how these and other modelling aspects have been applied in recent literature, and identify trends, methodological biases, and gaps, we conducted a scoping review following the PRISMA-ScR guidelines. Methods section details the internal protocol, eligibility criteria, search strategy (databases consulted, 2019 – 2024), screening procedures, and structured data-extraction form used for the included articles, which organized the information into the three categories employed in the analysis. These methodological procedures support the presentation of results in Results section.

## Methods

The study adhered to the guidelines and checklist of the Preferred Reporting Items for Systematic Reviews and Meta-Analyses extension for scoping reviews (PRISMA-ScR) [43]. All items from the PRISMA-ScR checklist were considered in this review, except for the critical appraisal of individual sources. This decision reflects the principal goal of our study, which is to map the literature on the mathematical modelling of AMR and to identify research gaps to inform future research directions, rather than to assess the methodological quality of each included study.

### Protocol and registration

No specific protocol was developed or registered prior to conducting this review. Although the creation and registration of a protocol are widely recommended to enhance transparency, reduce duplication of efforts, and improve reporting quality [44], this study did not have a formally registered protocol publicly available or accessible upon request. To ensure methodological rigor, however, an internal protocol was developed and adhered to by the research team, serving as a framework to guide the planning, conduct, and reporting of the review. The methodological approach followed the Arksey and O’Malley framework for scoping reviews [45], which was subsequently refined by Levac et al. [46] and by the Joanna Briggs Institute (JBI) guidance [47]. Future reviews building on the present findings may benefit from formal protocol registration on platforms such as open science framework (OSF), which accommodates a broader range of research designs, including scoping reviews.

### Eligibility criteria

We followed the PCC (Population, Concept, Context) framework recommended by the JBI [47] to guide the objective, research question, and inclusion criteria. An overview of the literature on the mathematical modelling of AMR is the scope of this review, with the search and selection of articles prioritizing studies that develop or apply mathematical models to describe the emergence, spread, and control of drug-resistant microbial populations. Based on the PCC framework, the following inclusion rules were defined:

1. *Population (P)*: studies applying AMR modelling to humans, animals, in vitro systems, combinations of these, or general contexts; the pathogens considered included bacteria, viruses, fungi, and parasites.
2. *Concept (C)*: studies employing dynamic mechanistic models to investigate AMR and its mechanisms. By mechanistic models, we refer to mathematical formalisms that explicitly represent biological and epidemiological processes (e.g., transmission, mutation, selection) to simulate system behaviour. Consequently, we excluded purely statistical models (e.g., regression models like GLM/GAM), time-series models (e.g., ARIMA, SARIMA), and artificial intelligence/machine learning models (e.g., neural networks, random forests) that are primarily data-driven and focus on correlation or prediction rather than on simulating underlying causal mechanisms.
3. *Context (C)*: studies conducted in intrahost, community, hospital/ICU, laboratory, or general settings, indexed in Scopus, Web of Science, or PubMed, published in English between January 1, 2019, and December 31, 2024.

The exclusion criteria included studies related to AMR without a formal mathematical structure or lacking a dynamic modelling component, articles that did not develop original models (e.g., purely descriptive works or statistical analyses without population dynamics), book chapters, narrative or systematic reviews without a new model proposal, conference abstracts, non-peer-reviewed material, papers published in languages other than English, and studies whose full text was unavailable. The period (2019 – 2024) was selected to capture recent methodological advances, reflect the continuous growth of the literature in the area, and allow for an updated analysis of the main trends in the mathematical modelling of AMR. Furthermore, while we acknowledge the importance of microbiome-level interactions in the emergence and establishment of AMR studies focusing exclusively on the gut microbiota or on within-host ecological dynamics, studies without explicitly modelling resistant pathogen populations and their transmission were excluded. Our focus was on mechanistic models that explicitly represent AMR dynamics in pathogenic populations, either at the within-host or between-host level, rather than models centred on commensal microbial communities or ecological networks unrelated to specific pathogens.

### Information sources

We searched Scopus, PubMed, and Web of Science for relevant literature, selecting these databases for their extensive coverage of mathematical modelling studies and rigorous indexing standards. The search was conducted between 1 January 2019 and 31 December 2024 and used a single Boolean string, adapted for each database, combining terms related to AMR (e.g., antimicrobial, antibiotic, antiviral, antifungal, antiparasitic resist*), resistance-transfer mechanisms (e.g., mutation, horizontal gene transfer, plasmid, conjugation, transduction, phage), and modelling approaches (e.g., mathematical model, compartmental model, agent-based model, stochastic model). All the search results were exported in CSV format, and duplicates were automatically removed via Python script available in our GitHub repository (see Appendix Supplementary Material). Two reviewers independently screened all titles and abstracts for eligibility, resolving discrepancies by consensus, with a third reviewer consulted when necessary. The reference lists of the included studies were also examined manually to identify additional relevant papers. Grey literature (e.g., preprints, dissertations, and technical reports) was excluded to maintain a focus on peer-reviewed research.

### Screening process

**Fig. 2:**
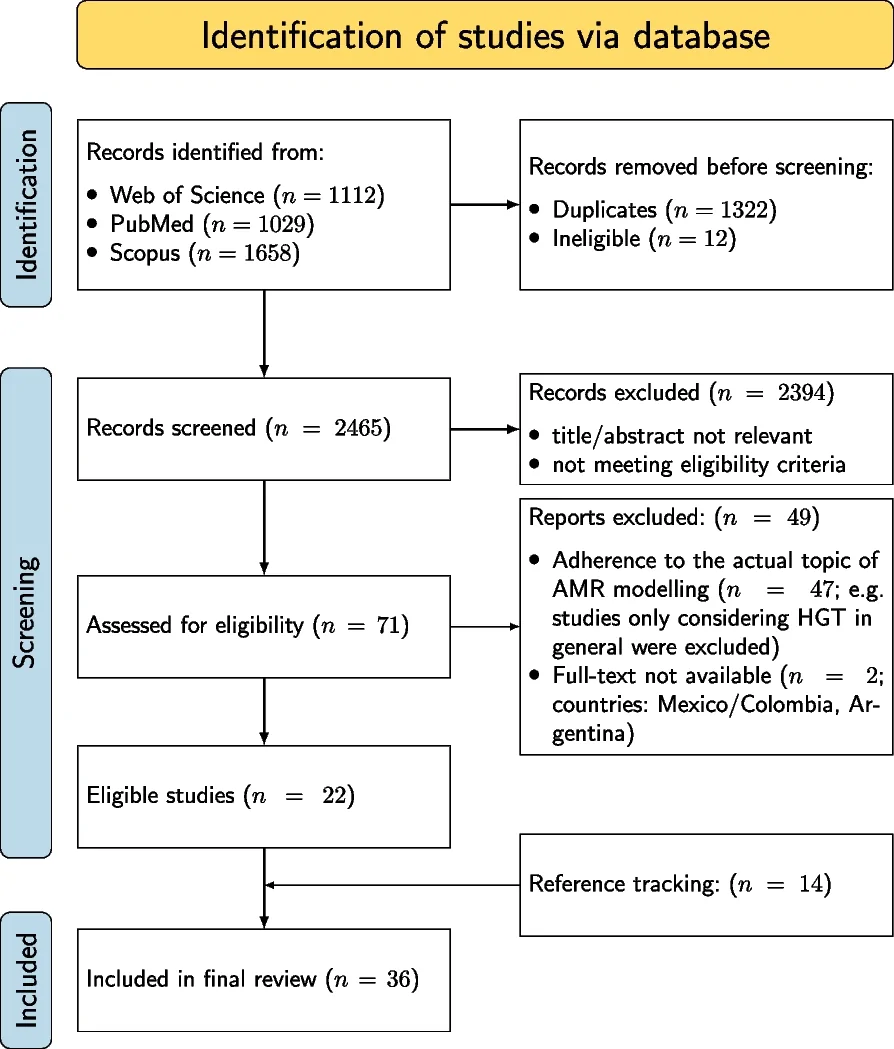
PRISMA flow diagram of the study selection process

The complete screening process is shown in Fig. 2. The searches were conducted in three databases on April 11, 2025, using the following terms:

- *Web of Science search*: ALL=((antimicrobial OR antibiotic OR antiviral OR antifungal OR antiparasitic)resist* AND (mechanism* OR mutation* OR ‘horizontal gene transfer’ OR plasmid OR transformation OR conjugation OR transduction OR phage) AND (math* OR dynamic* OR stochastic OR deterministic OR compartmental OR ‘agent-based’) model*), 1112 results.
- *PubMed search*: (antimicrobial OR antibiotic OR antiviral OR antifungal OR antiparasitic)resist* AND (mechanism* OR mutation* OR ‘horizontal gene transfer’ OR plasmid OR transformation OR conjugation OR transduction OR phage) AND ((math* OR dynamic* OR stochastic OR deterministic OR compartmental OR ‘agent-based’) model*), 1029 results.
- *Scopus search:* TITLE-ABS-KEY((antimicrobial OR antibiotic OR antiviral OR antifungal OR antiparasitic) AND (resist* OR tolerance OR persistence) AND (mechanism* OR mutation* OR ‘horizontal gene transfer’ OR plasmid OR transformation OR conjugation OR transduction OR phage) AND (math* OR dynamic* OR stochastic OR deterministic OR compartmental OR ‘agent-based’) AND model*) AND PUBYEAR > 2018 AND PUBYEAR < 2025, 1658 results.

Our study selection process, involving the identification, screening, and inclusion of relevant papers, is summarized in Fig. 2. Initially, we retrieved 3.799 articles, of which some 1.322 were discarded for duplication and some 12 were discarded because they were ineligible. The duplicate removal process was performed in Python, based on the DOI and title. All the articles exported from the three databases are available in the all_data folder in the GitHub repository (see Appendix Supplementary Material). One author read the title and abstract of the remaining 2.465 articles and, based on the research objectives, selected 71 articles using the pre-established eligibility criteria in the Eligibility criteria section.

These 71 studies were then independently evaluated by a second author, who reviewed the full texts. This step resulted in the exclusion of 49 studies. In particular, two studies were excluded because the full text could not be accessed; both were behind paywalls. The remaining 47 studies were excluded due to non-adherence to the actual topic of mechanistic mathematical modelling of antimicrobial resistance (e.g., studies only considering general horizontal gene transfer). The primary reasons were non-adherence to the core topic of AMR mechanistic modelling (e.g., models of general HGT not applied to AMR) and unavailability of the full text. Finally, to ensure comprehensiveness within the predefined temporal scope *(2019-2024)*, we performed backwards reference tracking on the 22 included studies. Only studies meeting the same eligibility criteria, including the temporal restriction, were considered. This process identified an additional 14 eligible studies, resulting in a final total of 36 studies for qualitative analysis in this review. The time frame (*2019-2024*) was intentionally defined to capture recent methodological developments in mechanistic mathematical modelling of antimicrobial resistance, rather than to provide a historical overview of the field.

### Information extracted from the included papers

We developed a data charting form to record the variables relevant to the scope of the study. Following the recommendations of Levac et al. [46], the form was iteratively updated as the team conducted the review and analysis of the included studies *(n = 36)*. To ensure objectivity and comparability across the 36 included studies, the data charting form was structured using closed questions. The Possible Values presented in Table 1 represent the predefined categorical options used by the reviewers during the extraction process to standardise the analysis and facilitate the subsequent thematic synthesis.

**Table 1:** Summary of categories and attributes extracted from the selected models

Model category	Recorded information	Signification	Possible values
Type and Context	Model type	Model type used in the study	Deterministic OR Stochastic OR Both
	Mathematical structure	Mathematical structure of the model	ODEs OR PDEs OR SDEs OR IDEs
	Host	Type of host represented in the model	Human OR Animal OR In-Vitro OR Human and Animal OR General OR Host Not Specified
	System context	System setting represented in the model	Within-Host OR Community OR Hospital OR Laboratory OR General OR Hospital/ICUs Or Within-Host and Between-Host OR Between-Host OR One-Health
	Pathogen category	Pathogen category represented in the model	Virus OR Bacteria OR General
	Specific pathogen	Specific pathogen(s) included in the model	*P. aeruginosa* OR *E. coli* OR *S. aureus* OR *A. baumanni* OR *S. pneumoniae* OR *C. difficie* OR *K. pneumoniae* OR HIV OR Combination OR Pathogen Not Specified
Construction and Correlated Parameters	Acquired resistance	Mechanism of resistance acquisition modelled	Mutation, HGT (conjugation/transformation), Recombination, Selection, Transmission, or Not explicitly modelled (alone or in combination)
	PK/PD parameters	Type of Pharmacokinetic/Pharmacodynamic (PK/PD) component included in the model	PK/PD OR PK OR PD OR PK/PD Not Modelled
	Spatial dispersion	Spatial consideration in the model	Yes OR No
	Fitness cost	Fitness cost for the growth of resistant strains	Yes OR No
	Immune response	Presence of host immune response in the model	Yes OR No
	Multiple resistance	Consideration of multiple AMR resistance in the model	Yes OR No
	Drug concentration	Threshold drug concentration for antimicrobial efficacy (e.g., MIC)	Yes OR No
Outputs and Validation	Treatment strategy	Antimicrobial treatment strategy in the model	Monotherapy OR Polytherapy OR Both OR Treatment Not Modelled
	Data Source	Source of data or parameter values used in the model	Literature, Experimental, Clinical, Simulated, Assumed, Estimated, or Calibrated (alone or in combination)
	Sensitivity analysis	Impact of parameters on model outputs	Yes OR No
	External validation	Compare predictions with other models/studies	Yes OR No
	Evolution of resistance	Changes in resistance over time within the model	Yes OR No
	Economic impact	Economic impact related to AMR	Yes OR No
	Practical applications	The model results can be used in practical applications	Yes OR No

The three categories analysed contain relevant information for understanding how mathematical models are developed and applied to the study of AMR. Specifically, the data in Table 1 encompass (i) the epidemiological and biological context of the model (Type and Context); (ii) the structural and mechanistic aspects involved in model construction (Construction and Correlated Parameters); and (iii) the analytical strategies and evaluated results (Outputs and Validation). These elements provide an overview of how modelling studies address the complexity of AMR systems. The subsequent Figs. 5, 6, 7, 8 and 9 visually summarise the frequency of these categories across the 36 reviewed studies, offering a clear picture of prevailing trends and under-explored areas in the field.

The first category, Type and Context, addresses the research setting in which the model is defined. It includes the mathematical formulation (e.g., deterministic or stochastic), the model structure (e.g., ODEs or PDEs), and the host type under consideration (e.g., human, animal, or in vitro). It also integrates the broader epidemiological setting (e.g., hospital, community or intra-host) and the pathogens addressed, whether at the category level (e.g., bacteria or virus) or at the level of specific organisms (e.g., *E. coli*, *S. aureus*). These characteristics define the validity domain of the model and establish the biological and epidemiological context for interpreting its results.

The second category, Construction and Correlated Parameters, encompasses the internal mechanisms and biological assumptions implemented in the models. It records how resistance is represented (mutation, horizontal gene transfer), whether pharmacokinetic/pharmacodynamic (PK/PD) processes are explicitly included, and whether spatial dispersion is modelled. Additional elements include host immune-response dynamics that act on pathogen clearance, fitness costs for resistant strains, the possibility of multiple resistances, and explicit drug concentration thresholds (e.g., MICs). Together, these characteristics determine the structural complexity of the models and influence their ability to capture biological realism.

The third category, Outputs and Validation, focuses on the strategies employed to analyse and test the models. It includes whether treatment strategies are simulated (e.g., monotherapy, polytherapy), data sources used to inform model structure or parameterisation (e.g., literature, experimental, or clinical data), whether sensitivity analyses are performed to assess parameter influence, and whether external validation is attempted. The outputs considered may include predictions of resistance evolution, evaluation of economic impacts, or the identification of practical applications for policy or clinical decision-making. These aspects provide insight into the robustness, applicability, and translational potential of the modelling frameworks.

Together, these three categories, summarized in Table 1, provide a framework that captures the epidemiological and biological context, the structural mechanisms of the models, and the analytical results of the studies, including whether the model incorporated an economic component (e.g., cost or cost-benefit considerations) when applicable. The structured data presented in this table formed the basis for the next steps of the thematic and quantitative analysis, allowing the identification of trends, gaps, and clusters in the existing literature on mathematical modelling of AMR covered in this scoping review. The detailed list of aspects extracted from each study can be found in Appendix Supplementary Material. The file questionnaire_answers.csv, containing all the extracted elements from the 36 studies included in this review, is available in our GitHub repository (Appendix Supplementary Material).

## Results

**Fig. 3:**
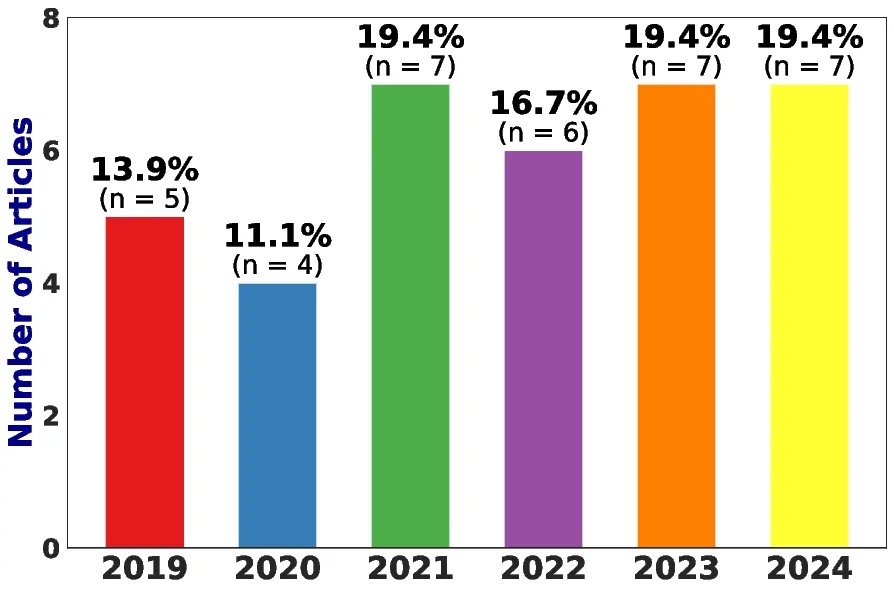
Temporal distribution of the 36 studies included in the scoping review (2019–2024). Illustrating a consistent research output in the mathematical modelling of AMR over the period

A total of 36 studies were included in this scoping review [42, 48–82]. The period from 2019 – 2024 was selected to capture the most recent developments in the mathematical modelling of AMR, reflecting the increasing interest and methodological diversification observed in recent years. The temporal distribution of the publications is shown in Fig. 3. Five articles *(13.5%)* were published in 2019, four *(10.8%)* in 2020, and eight *(21.6%)* in 2021. Six studies *(16.2%)* were published in 2022, while the most recent years, 2023 and 2024, each contributed seven articles *(18.9%)*. This distribution highlights a consistent research effort in modelling AMR dynamics during the period considered.

**Fig. 4:**
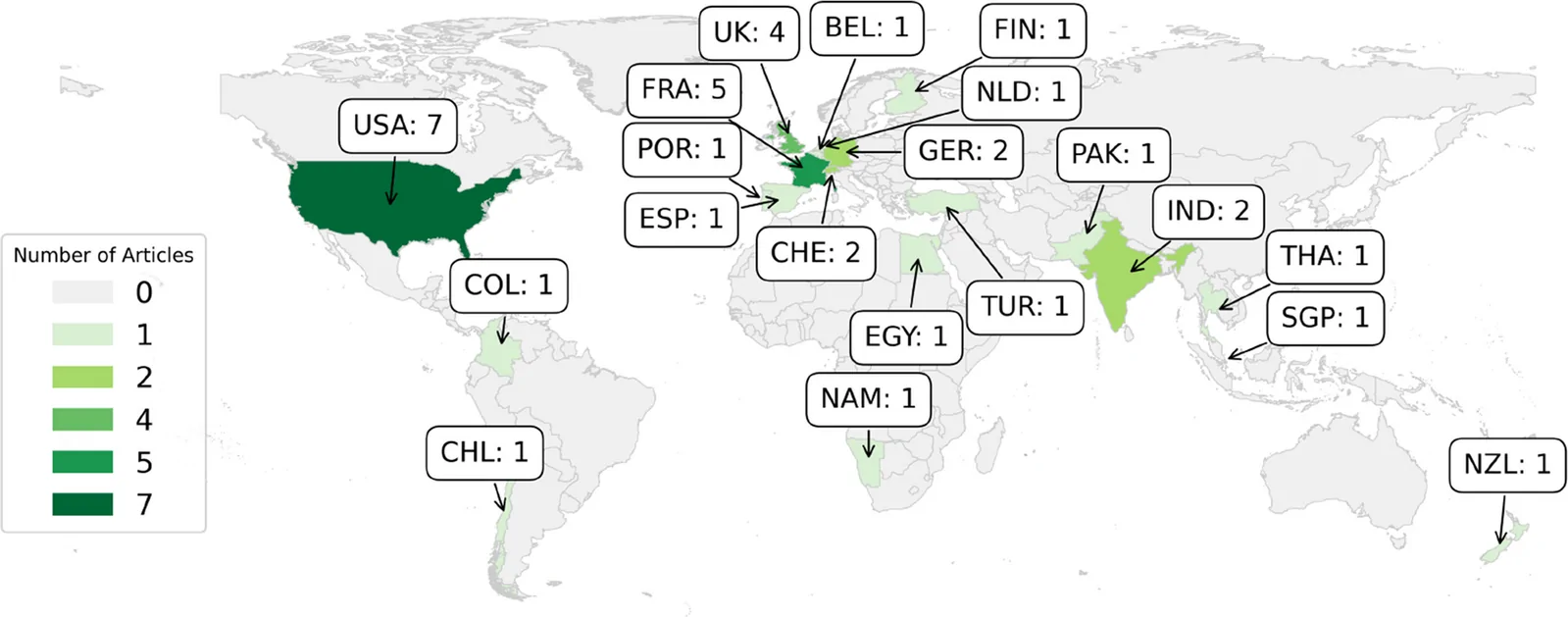
National affiliation of the first author’s institution for articles included in the scoping review. The figure highlights the predominance of studies from high-income countries (HICs) and the underrepresentation of contributions from the Global South

The 36 included studies were affiliated with institutions from 20 different countries. The most represented were the United States (seven studies), France (five), and the United Kingdom (four). Several other high-income countries contributed fewer studies, including Germany and Switzerland (two each). Portugal, Belgium, the Netherlands, New Zealand, Singapore, Spain, Finland, and Turkey (one each). Countries from the Global South, such as India (two studies), Egypt, Pakistan, Colombia, Chile, Namibia, and Thailand, were underrepresented, with only one study each. This distribution highlights a clear geographical imbalance in research on mathematical modelling of AMR, with a predominance of high-income countries (HICs) (see Fig. 4). However, as discussed in section Discussion, this predominance appears to be specific to the niche of mechanistic mathematical modelling and does not necessarily reflect leadership patterns observed in other domains of AMR research.

### Model type and context

The reviewed studies were first categorized on the basis of their methodological approach and the epidemiological context they aimed to represent. In particular, we examined the mathematical formulation of the models (e.g., deterministic, stochastic, etc), as well as the epidemiological scales represented. This analysis highlights a clear predominance of certain model types and mathematical structures, choices that are often influenced by the research question, the extent and precision of available data, and the necessity to reconcile analytical tractability with computational feasibility. Moreover, the distribution of host organisms, system contexts, and pathogen types offers valuable insights into the main priorities and limitations guiding current research. These categories provide a comprehensive view of how modelling efforts have been framed in the AMR field, showing prevailing practices and underexplored directions.

**Fig. 5:**
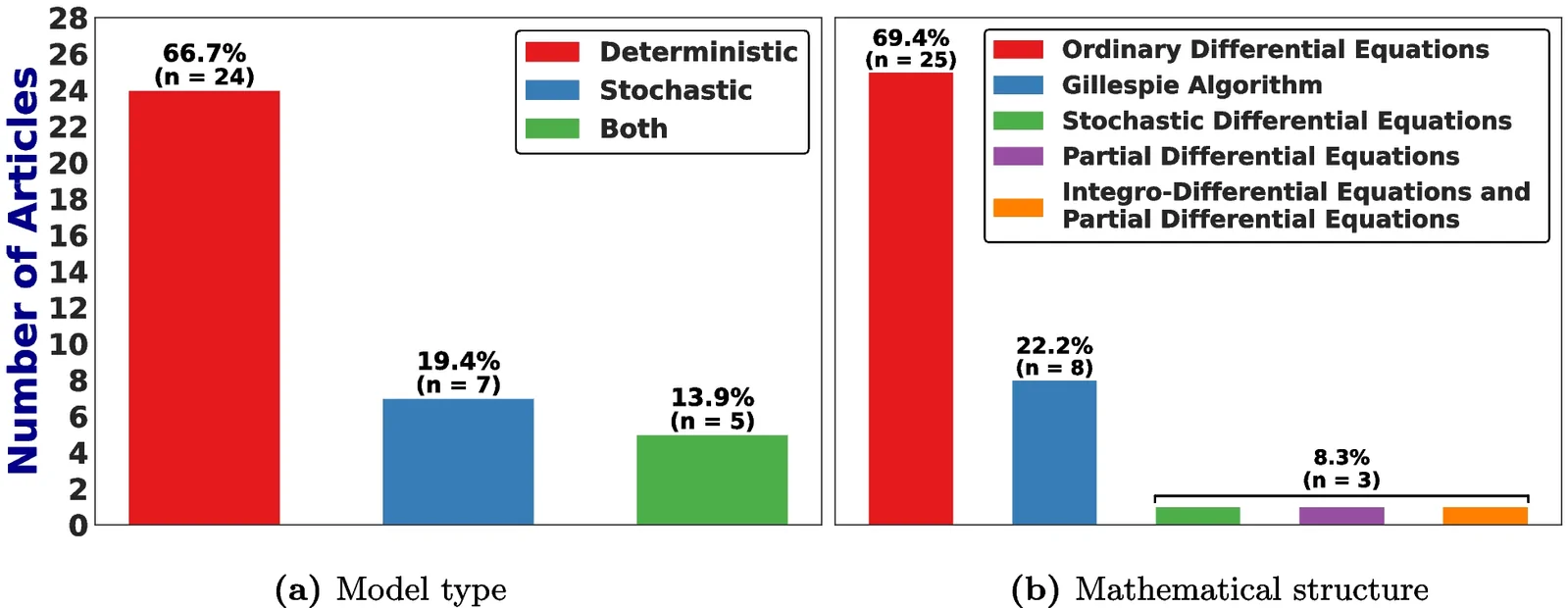
Overview of modelling approaches in the reviewed studies. **a** Shows the distribution of model types. The strong preference for deterministic models *(66.7%)*, which calculate a single, average outcome, is evident. In contrast, stochastic models *(19.4%)*, which incorporate randomness and are crucial for capturing rare events like the initial emergence of resistance, are used much less frequently. **b** Details the specific mathematical structures employed. The dominance of ordinary differential equations (ODEs) *(69.4%)* reflects their analytical tractability. More complex structures like stochastic differential equations (SDEs), partial differential equations (PDEs), and integro-differential equations (IDEs), which can model randomness and spatial variation, are rarely used, indicating a potential gap in capturing real-world complexity

Figure 5 illustrates the results of the 36 studies related to the type of model and the mathematical structure used. Figure 5a presents the distribution of model types adopted in the reviewed studies, highlighting that twenty-four articles *(66.7%)* utilised deterministic models [42, 49–51, 53–56, 58–60, 63, 65, 67, 71–73, 75–80, 82], seven studies *(19.4%)* analysed stochastic models [48, 62, 64, 68–70, 81] and five *(13.9%)* conducted analyses and comparisons between both models [52, 57, 61, 66, 74]. Regarding the nature of the randomness, most stochastic models focus on demographic stochasticity, capturing fluctuations in population size derived from the discrete and probabilistic nature of individual events such as births, deaths, and mutations [52, 57, 61, 62, 64, 66, 68–70, 74, 81]. These studies typically employ the Gillespie algorithm or branching processes to model the survival probability of rare resistant mutants in finite populations. In contrast, environmental stochasticity is primarily addressed in [48], which models the intrinsic growth rate as a random variable (Gaussian white noise) to simulate rapid changes in the external environment. Notably, [48] also recognises that antimicrobial drugs can simultaneously induce both demographic and environmental stochasticity. Now, for the mathematical structure (Fig. 5b) we find 25 studies *(69.4%)* that use ordinary differential equations (ODEs) [42, 49–56, 58–63, 65, 71–73, 75, 77–80, 82], eight articles *(22.2%)* adopted the Gillespie algorithm [57, 64, 66, 68–70, 74, 81], one study used stochastic differential equations (SDEs) [48], one used partial differential equations (PDEs) [67] and one utilized integro-differential equations (IDEs) and PDEs [76], each representing one *2.8%* of the studies. Among the articles that used Gillespie’s algorithm, [66, 69, 70, 74, 81] applied Gillespie’s exact algorithm. Articles [57, 64] used approximations, such as the *τ *-leap algorithm or its approximation, which is based on the binomial distribution [68]. Although article [81] uses Gillespie’s algorithm to simulate its birth–death model, it employs linear noise approximation (LNA), a technique that approximates discrete stochastic dynamics via an SDE for the purpose of moment discovery and analysis. The article [48] developed a primarily continuous approach, modelling the dynamics through an SDE and using the Euler–Maruyama method for numerical simulation.

The predominance of deterministic models in the reviewed literature, especially those based on ODEs, is frequently attributed in the broader epidemiological modelling literature to their relatively simple implementation, lower computational cost, and analytical tractability [83]. These models allow researchers to investigate the population-level dynamics of AMR and evaluate the effects of interventions on the basis of simplified representations of biological and epidemiological processes, which can be valuable when detailed data are scarce [26, 84]. In contrast, stochastic or high-dimensional models often require more detailed parametrisation and computational resources, making them less feasible in settings with limited or aggregate data [39, 85]. Moreover, software tools commonly used in mathematical epidemiology, such as MATLAB, R, and Python, are well-established numerical solvers for ODEs, facilitating their adoption. For example, MATLAB provides the ODE suite (ode45, ode15s, among others) [86], R includes packages such as deSolve for deterministic model implementation [87], and Python offers the SciPy.integrate module (solve_ivp, odeint) for flexible ODE solving [88]. This methodological accessibility reinforces the preference for ODE-based approaches, even in complex problems such as AMR. Nevertheless, this trend may also reflect limitations in available data, as more complex models (e.g., stochastic or spatial) typically require finer-grained information that is often unavailable or difficult to obtain in AMR surveillance contexts [89, 90].

Although ODE-based models offer ease of analysis, the strong dependence on these deterministic structures still imposes an important methodological limitation for public health applications. The emergence and spread of AMR, particularly the emergence of resistance (rare events) or rapid changes in small populations (e.g., in hospitals), are essentially stochastic processes. The scarcity of models that capture stochasticity (only *19.4%*) reduces the predictive power of analyses and compromises their usefulness in complex and realistic epidemiological contexts. This demonstrates the need for current modelling practices to capture real-world dynamics and effectively respond to practical challenges.

**Fig. 6:**
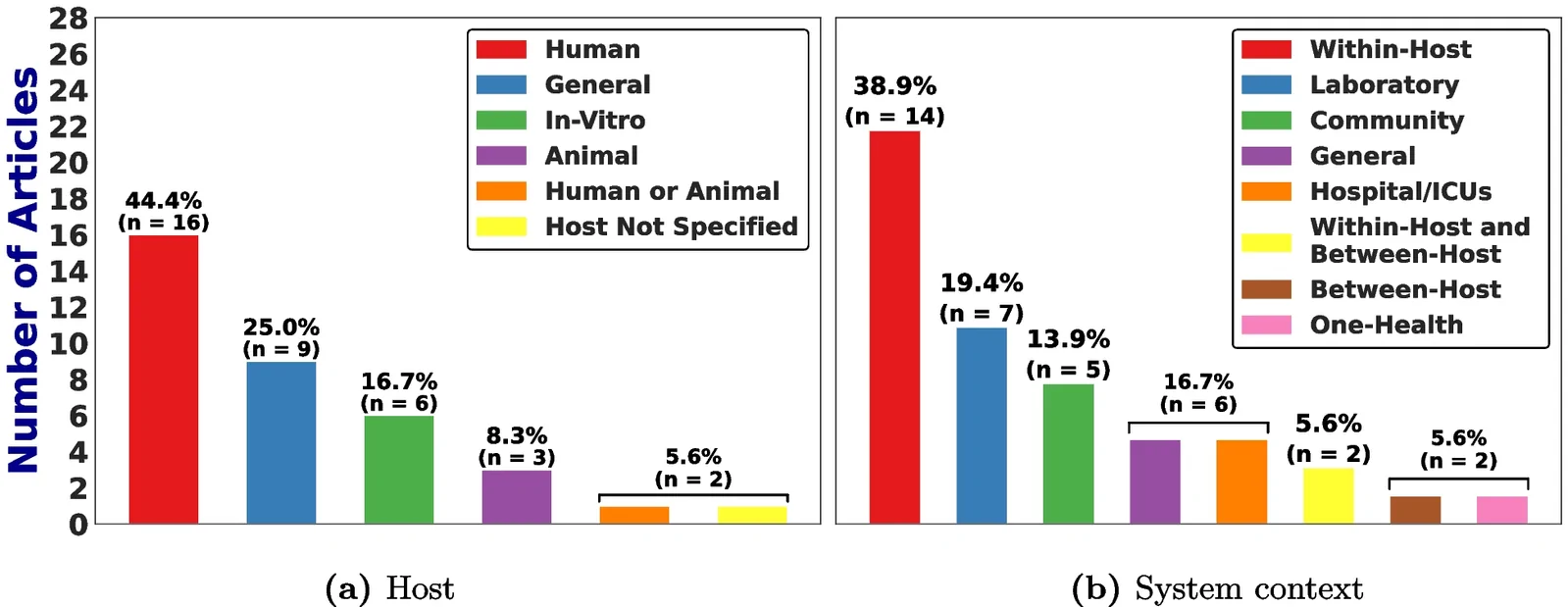
This figure illustrates the host organisms and epidemiological contexts represented in the selected studies. **a** Shows the type of host modelled. The overwhelming focus on the human host *(44.4%)* highlights the clinical priority of AMR. Notably, models including animals *(8.3%)* are scarce, and a true One Health approach, integrating human, animal, and environmental compartments, is virtually absent (only one study). **b** Presents the system context. A significant portion of studies focus on within-host dynamics *(38.9%)*, exploring bacterial evolution during treatment. However, hospital *(8.3%)* and community *(13.9%)* transmission models, essential for infection control, are underrepresented

Figure 6 provides an overview of the host organisms modelled and the system-level settings explored in the selected studies. As shown in Fig. 6a, human hosts were the most common, used in nineteen studies *(44.4%)* [42, 51, 52, 55–57, 61, 64, 69, 73, 75–79, 82], the general host appeared in nine articles *(25%)* [48, 49, 54, 58, 59, 62, 67, 70, 74], in vitro appeared in six studies *(16.7%)* [53, 65, 66, 68, 80, 81], three articles *(8.3%)* [60, 71, 72] used models that can be applied to animals; one study *(2.8%)* [50] included both humans and animals, and one article [63] did not focus on a specific host. With respect to the modelled epidemiological context (Fig. 6b), more than one-third of the studies (14/36) focused on within-host dynamics [42, 48, 49, 51, 52, 54, 58–62, 69, 72, 79]. Laboratory settings were represented in seven studies *(19.4%)* [53, 65, 66, 68, 71, 80, 81]. Five studies *(3.9%)* examined community-level contexts [55, 56, 63, 64, 73]. Three studies *(8.3%)* addressed general modelling frameworks [67, 70, 74]. The other three studies *(8.3%)* focused on hospital/ICU environments [77, 78, 82]. Two studies *(5.6%)* combined within- and between-host scales [57, 76]. One study *(2.8%)* investigated between-host dynamics [50] and one study focused on One-Health contexts [75].

These results highlight the strong predominance of models focused on human hosts, emphasizing the clinical priority of AMR in public health contexts [4]. Additionally, the low representation of animal hosts and the absence of models that include several sectors (One Health) suggest an important gap, with recent reviews highlighting that multi-scalar models are rare, given their great potential to capture crucial characteristics that drive AMR [91]. In general, these studies reveal a disconnected scenario, reflecting both limited multidisciplinarity and the absence of data across human, animal, and environmental sectors [92]. Future mathematical models should use multi-scalar and integrated approaches, adopting the One Health perspective, and explicitly connect human hosts, animals, and environments to effectively capture the complex ecology of AMR [93, 94].

**Fig. 7:**
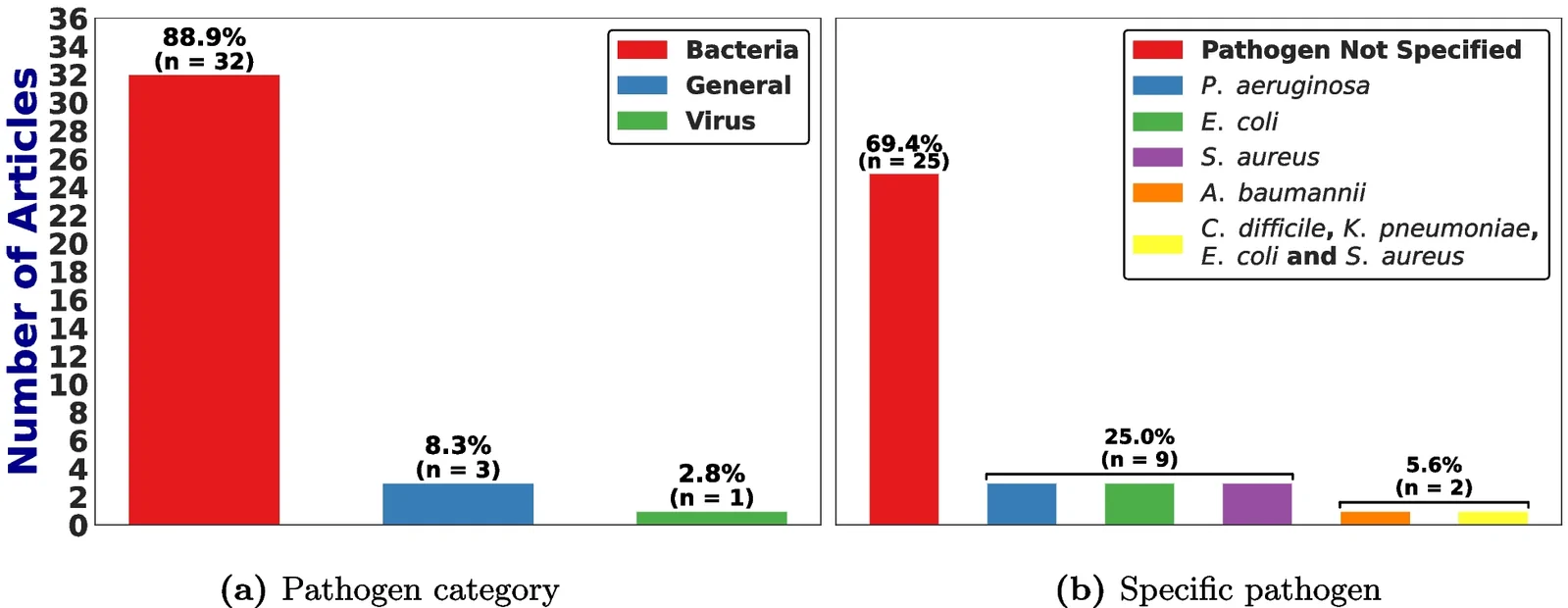
Pathogens targeted by AMR models. This figure summarises which microorganisms are the focus of modelling efforts. **a** Demonstrates a near-exclusive focus on bacteria *(88.9%)* , which aligns with the major global burden of bacterial AMR. However, it also reveals a critical gap, as AMR in viruses, fungi, and parasites is practically unmodelled, despite its growing clinical importance. **b **Lists the specific pathogens modelled. A majority of studies (25/36) did not specify a pathogen, opting for a general representation. When specified, the models target WHO priority pathogens like *Pseudomonas aeruginosa*, *Escherichia coli*, and *Staphylococcus aureus*, confirming that research priorities are aligned with public health threats

Figure 7 summarizes the types of pathogens addressed in the selected studies. As shown in Fig. 7a, the vast majority (32/36) of the models focused on bacteria [42, 48–51, 53–56, 58–69, 71–73, 75–82], whereas only one study *(2.8%)* addressed a viral infection [52], and another three *(8.3%)* adopted a general pathogen category without specifying the type [57, 70, 74]. In terms of specificity (Fig. 7b), a significant portion of the studies (25/36) did not explicitly model a specific pathogen (no specific pathogen) [42, 48–53, 55–64, 67, 69, 70, 73–76, 81]. Among the studies that reported the pathogen, the most frequently modelled bacteria were *Pseudomonas aeruginosa*, addressed in three studies [71, 72, 77], *Escherichia coli*, examined in three studies [65, 66, 68], and *Staphylococcus aureus*, modelled in three studies [54, 79, 80]. Other pathogens included *Acinetobacter baumannii*, in a single study [82], and a composite group comprising *Clostridioides difficile*, *Staphylococcus aureus*, *Escherichia coli* and *Klebsiella pneumoniae*, in a single study [78].

### Model construction and correlated parameters

This section examines the model parameterisation, highlighting which biological complexities are commonly integrated and which are frequently omitted, potentially pointing to important gaps in the current modelling landscape. In particular, it considers the representation of resistance acquisition mechanisms, the incorporation of PK/PD processes, and the inclusion of additional biological features such as fitness costs, host immune responses, spatial dispersion, and multiple resistance mechanisms.

**Fig. 8:**
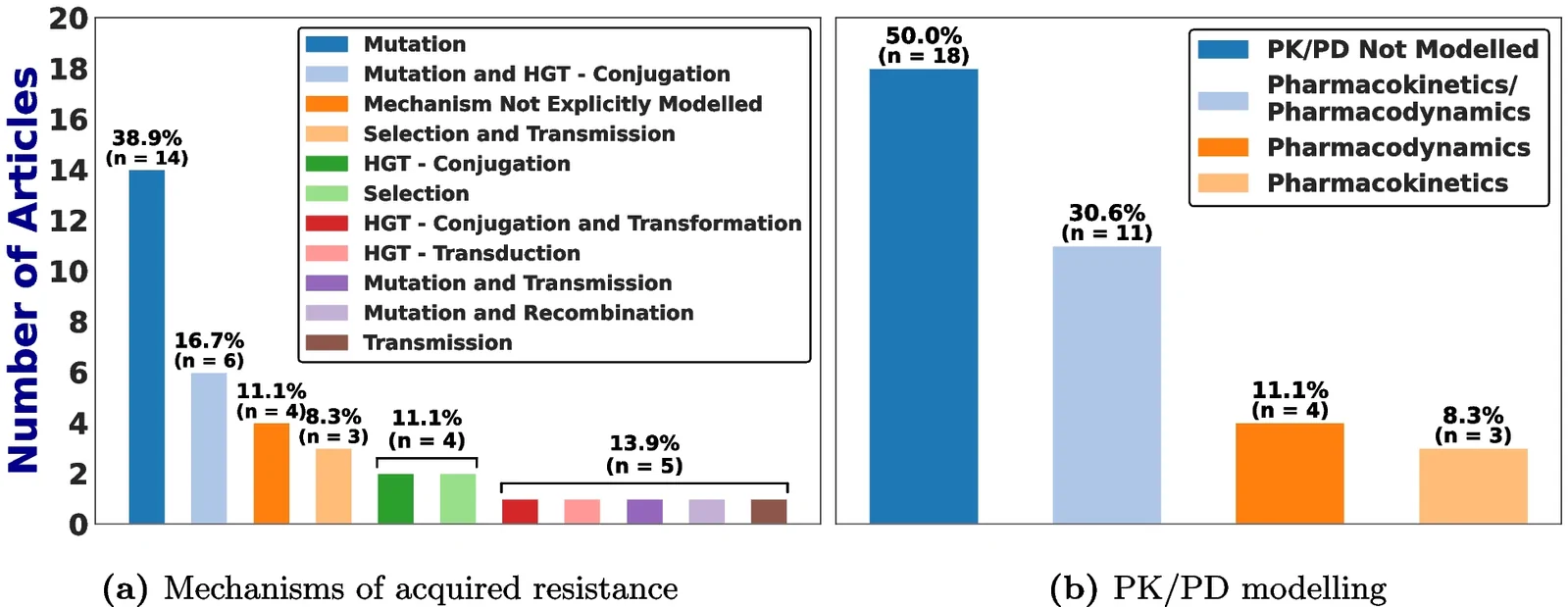
Representation of biological mechanisms in AMR models. **a** Shows the genetic mechanisms by which bacteria acquire resistance. Mutation *(38.9%)* and conjugation (a type of horizontal gene transfer – HGT) are the most modelled pathways, as they are well-characterised. However, other important HGT mechanisms like transformation and transduction are almost entirely neglected, potentially oversimplifying the spread of resistance genes in natural environments. **b** Indicates whether models include pharmacokinetic/pharmacodynamic (PK/PD) relationships, which link drug concentration to its killing effect. Half of the studies *(50%)* did not include any PK/PD component, limiting their ability to inform optimal drug dosing regimens. Full PK/PD integration was present in only one *30.6%* of studies

Figure 8 shows the results regarding resistance acquisition mechanisms and the use of PK/PD modelling in the thirty-six articles included in this review. In particular, regarding the mechanisms of resistance acquisition (Fig. 8a), a total of 14 studies *(38.9%)* considered mutation as the sole mechanism [52, 54, 55, 57, 61, 62, 65, 68, 70–72, 74, 76, 81]. Six studies *(16.7%)* reported a combination of mutation and HGT via conjugation [51, 58–60, 64, 79], whereas four studies *(11.1%)* did not explicitly model a specific resistance acquisition mechanism [48, 49, 63, 66], three articles *(8.3%)* considered selection and transmission [50, 56, 77], two *(5.6%)* considered HGT via conjugation exclusively [42, 78], two focused exclusively on selection [67, 73]. Other acquisition-related categories with one study *(2.8%)* each included HGT via conjugation and HGT via transformation [53], HGT via transduction [80], mutation and transmission [69], mutation combined with recombination [75], and transmission [82]. Figure 8b summarizes how PK/PD modelling was incorporated in selected studies. Half of the studies (18/36) focused on resistance dynamics without incorporating PK/PD modelling [48, 50, 51, 56–60, 63, 64, 67, 70, 73, 75–78, 82], 11 studies *(30.6%)* combined PK/PD modelling in their analyses [42, 49, 52–54, 61, 62, 66, 71, 72, 80], four articles *(11.1%)* used only PD modelling [68, 69, 74, 81], and three studies *(8.3%)* focused on PK modelling [55, 65, 79].

Mutation and HGT via conjugation are frequently modelled as the primary routes of resistance acquisition because they are specific, quantifiable, and relatively straightforward to incorporate into mathematical frameworks [95, 96]. In contrast, HGT via transformation or HGT via transduction, while crucial in the spread of resistance, are more complex processes to model, involving factors such as exogenous DNA uptake (transformation) or viral infection (transduction) [97, 98]. The lack of PK/PD data in many studies is partly due to the focus on the population dynamics of resistance, without explicit modelling of drug–host interactions. Furthermore, PK/PD modelling requires detailed experimental data, which are often unavailable or not the objective of the study, leading to a preference for simpler approaches [99, 100].

Table 2 summarizes several important features concerning model construction and correlated parameters identified across the included studies. Most of the reviewed studies *(28/36; 77.8%)* reported a fitness cost associated with the resistant strain [42, 51–62, 65, 66, 69–81]. In contrast, other important features were largely absent, and only four studies considered spatial dispersion [66, 67, 73, 75], and just eight included the host immune response [49, 51, 55, 57, 71, 72, 76, 79]. The modelling of multiple resistance, although relevant to the biological reality of AMR, has been performed in only eight studies [54, 59, 61, 64, 67, 75, 78, 80]. Drug concentration, an essential element for capturing PK/PD processes, was explicitly incorporated as a dynamic variable in 18 of the 36 studies analysed [42, 49, 52–54, 61, 62, 65–72, 74, 80, 81]. The remaining studies modelled treatment effects through simplified or empirical functions without including antibiotic concentration explicitly.

**Table 2:** Summary of the presence or absence of model characteristics within the construction and correlated parameter categories across the 36 studies reviewed

Presence	Biological Mechanism				
	Spatial dispersion	Fitness cost	Immune response	Multiple resistance	Drug concentration
Yes	4	28	8	8	18
No	32	8	28	28	18

These characteristics presented in Table 2 reveal important trends and limitations in the current practices of mathematical modelling of AMR. The frequent incorporation of fitness costs in models reflects their analytical tractability and solid foundation in the evolutionary theory of AMR [101, 102]. On the other hand, the host immune response is a central component in clearing infections and resistance, yet it is often neglected [103]. A robust immune response can reduce the required duration of treatment and even alter the mutant selection range by limiting the drug concentration that favors partially resistant strains [104]. Moreover, spatial dispersal has rarely been considered, despite being a key factor in the spread of resistance in heterogeneous environments, where host mobility, variability in treatment access, and unequal drug distribution create complex selective landscapes [105, 106]. Additionally, the modelling of multiple resistance genes or mechanisms remains limited, despite its growing relevance in the face of emerging multidrug-resistant (MDR) and extensively drug-resistant (XDR) strains, as in the case of MDR/XDR tuberculosis [38, 107].

### Model outputs and validation

This subsection examines relevant aspects of model outputs and validation in the studies included in this review, such as the type of treatment used, which shapes the predicted selective pressures that drive resistance. Other important characteristics include the strategies adopted for model validation and outcome characterization, which demonstrate the robustness, applicability, and practical relevance of the modelling results. Analysing these aspects, we identify common practices, methodological preferences, and critical gaps (e.g., limited integration of economic impact) that prevent the models from comprehensively informing clinical and policy decision-making.

**Fig. 9:**
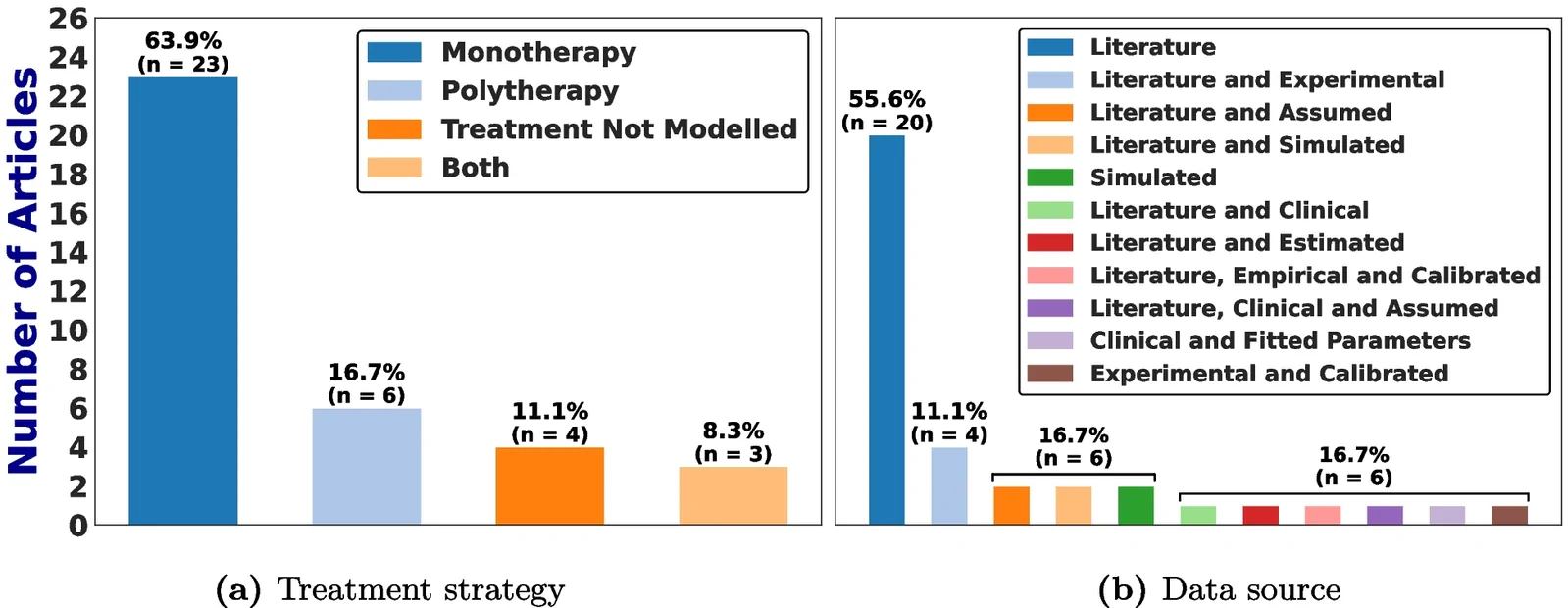
Treatment strategies and source of data used in the mathematical models included in the review. **a** Displays the types of antimicrobial therapy modelled. Monotherapy *(63.9%)* is the dominant strategy, reflecting both its common clinical use and the relative simplicity of modelling a single drug. The inclusion of polytherapy *(16.7%)*, which is crucial for managing multidrug-resistant infections, is limited. **b** Reveals the primary sources of data for model parameters. The vast majority of studies rely on literature-based parameters *(55.6%)*, which, while useful for theoretical exploration, may not capture context-specific dynamics. Only a small fraction of studies use direct experimental *(2.8%)* or clinical data *(2.8%)* for calibration, highlighting a potential gap between model development and real-world validation

Figure 9a shows the types of antimicrobial treatment modelled in the included studies. Notably, the vast majority of studies (23/36) adopted monotherapy [48, 49, 51–53, 55–58, 60, 62, 63, 65, 66, 68–70, 73, 74, 76, 77, 79, 81], six studies *(16.7%)* used polytherapy [54, 61, 67, 71, 72, 78], four studies did not model antimicrobial treatment [50, 59, 64, 82], and three studies *(8.3%)* included both strategies (monotherapy and polytherapy) [42, 75, 80]. Polytherapy in these models refers to the simultaneous use of two or more antimicrobial agents or the combination of an antimicrobial drug with an alternative therapeutic approach, such as bacteriophage therapy. Figure 9b presents the sources of data used to inform model development in the included studies. Most studies (20/36) relied exclusively on literature-derived parameters [42, 48, 49, 51–53, 57–61, 63, 64, 69, 70, 74–77, 79]. Four studies *(11.1%)* that combined literature and experimental data [66, 68, 71, 80]. Three distinct categories included two studies each *(5.6%)*: models based on literature and assumed parameters [54, 55], models combining literature information with simulated inputs [62, 72], and models relying exclusively on simulated data [67, 81]. The remaining categories were represented by a single study each *(2.8%)*, including models that combined clinical data, literature, and assumed parameters [50]; models integrating literature data with empirical calibration [56]; experimental data with calibration procedures [65]; literature and clinical data [73]; literature-derived and estimated parameters [78]; and clinical data with fitted parameters [82].

The frequent use of monotherapy in the included studies may reflect both practical and methodological factors. From a clinical point of view, monotherapy remains the standard treatment approach for many bacterial infections, particularly in contexts where resistance to first-line drugs is still limited or where surveillance systems are insufficient to guide combination therapy [89, 108, 109]. In terms of modelling approaches, monotherapy simplifies the representation of treatment dynamics and reduces the number of parameters required, facilitating model calibration and analysis [110]. In addition, the lack of detailed empirical data on PK/PD interactions between drugs in combination regimens probably reduces the incorporation of polytherapy into mathematical models [111]. These factors combined contribute to the increased adoption of monotherapy in mathematical models of AMR. The predominance of literature-based parameterisation suggests that many studies relied primarily on previously published estimates rather than direct calibration to empirical or clinical datasets, and although this strategy facilitates model construction and supports broader theoretical exploration, it may limit the capacity of models to capture context-specific resistance dynamics, with systematic assessments identifying validation and calibration as key methodological components whose implementation remains inconsistent across studies [112].

Table 3 summarizes relevant features concerning model outputs and validation identified across the review of included studies. A sensitivity analysis was performed in twenty-seven studies [42, 48, 50, 53, 55–59, 61, 62, 64–74, 77, 78, 80–82], external validation is included in twenty-six studies [42, 50, 52, 53, 55–62, 65–76, 78, 80], and almost all of the studies included evolution of resistance in their models, except for ten [48, 49, 51, 54, 63, 64, 77, 79, 81, 82]. On the other hand, although the economic impact of AMR is widely recognized, it is rarely incorporated into mathematical models, with only two studies explicitly including economic components in their formulations [56, 64]. Moreover, thirty-four studies assessed the practical application of interventions [42, 50–82]. While 34/36 studies discuss practical applications, the models’ structure and level of detail determine how useful these applications are for formulating policy. Because they make it possible to assess how treatment duration, frequency, and intensity affect bacterial suppression and the selection of resistant strains, models that incorporate PK/PD parameters are especially pertinent for directing prescribing policies. A significant barrier to converting findings into clinical recommendations for the management of microbial strains is indicated by the lack of these parameters.

**Table 3:** Summary of the presence or absence of model characteristics within the outputs and validation categories across the 36 studies reviewed

Presence	Output/Validation				
	Sensitivity analysis	External validation	Evolution of resistance	Economic impact	Practical applications
Yes	27	26	26	2	34
No	9	10	10	34	2

Furthermore, the ability to forecast the emergence and persistence of resistance under various therapeutic approaches is enhanced by the inclusion of evolutionary mechanisms, such as fitness costs linked to resistance or horizontal gene transfer. The realism of projections is further enhanced by taking individual heterogeneity, treatment compliance, and previous antimicrobial exposure. Additionally, the incorporation of economic factors like hospital expenses, duration of stay, or financial impact enhances the significance for public health choices, particularly in environments with limited resources. Therefore, even though the majority of research claims to have real-world applications, the ability to effectively inform public policies and clinical guidelines depends on the combined incorporation of specific factors that are still lacking in a sizable portion of the reviewed literature.

All the information in Table 3 is relevant when referring to the mathematical modelling of AMR. However, we highlight the scarcity of studies that incorporate the economic impact that AMR has on local and global economies. It is estimated that, by 2050, global financial losses associated with AMR could range from 300 billion to 1 trillion dollars [113], with accumulated losses of economic output in Organization for Economic Cooperation and Development (OECD) countries on the order of 20 – 35 trillion [3]. Moreover, the World Bank warns that AMR could increase poverty levels, with disproportionate effects on low- and middle-income countries (LMICs) [114]. In these countries, the higher costs of medical care are driven mainly by the need for prolonged treatment and additional hospitalizations. This reality is intensified by factors such as limited access to effective treatments, weak regulation, low awareness of the proper use of antibiotics, and inadequate dissemination of therapeutic guidelines [115]. The integration of economic dimensions is fundamental, without cost-effectiveness analyses, models fail to provide the quantifiable evidence needed for policymakers to justify investments in intervention programmes, especially in LMICs where the economic burden of AMR is most severe. The economic return, translated into reduced hospitalisations and increased productivity, must be an output for science to be converted into political action.

## Discussion

AMR represents one of the major contemporary global challenges to public health. Given the growing use of dynamic mathematical models to understand the mechanisms driving its emergence and spread, this scoping review synthesized evidence from 36 studies published between 2019 and 2024 that employed mathematical modelling to investigate different aspects of AMR. From the results, we identified significant trends, prevalent methodological approaches, and essential research gaps. During the period analysed in the review, the number of annual publications varied from four to seven, demonstrating sustained and developing research activity in AMR. The included studies originate from institutions in 20 countries, with the United States, France, and the United Kingdom representing the countries with the most publications, highlighting both the influence of English-language search criteria and the concentration of publications in countries where greater research funding is allocated.

This geographical imbalance likely reflects structural inequities in global knowledge production rather than differences in scientific capacity, as evidenced by persistent disparities in research funding, publication networks, and access to data across regions [116–118]. Despite this, the wider AMR research landscape does not conform to this pattern. Emerging economies, especially China, take the lead in areas such as environmental monitoring (Deza Cruz et al., 2025 [119]), highlighting how geographical leadership can differ according to the theme. On the other hand mechanistic mathematical modelling remains highly concentrated in rich countries which could be a function of differences clearly in modelling capacity, infrastructure, and already established theoretical traditions. The search strategy we employed in this scoping review might also account for this distribution because it focused on English journals and recent mechanistic studies (*2019-2024*), significantly older ones were not included. Therefore, we should take note of structural inequalities and the review characteristics when considering the imbalance. The predominance of studies included in this review being located in HICs can be attributed to several factors: (i) substantially greater investments in research and development (R&D) in HICs (averaging *2.5%*-$\$3\%$ of gross domestic product (GDP)) compared to most Global South nations (*<1%* of GDP), which enables advanced infrastructure for complex modelling research [116, 118]; (ii) publication biases in high-impact journals, where editorial boards and peer review networks remain predominantly concentrated in North America and Europe [117]; and (iii) resource-dependent barriers, including limited access to specialized software, epidemiological data repositories, and computational power in low-research-funding environments [120, 121]. Consequently, while our review includes valuable contributions from countries such as India and Egypt, research from these and other less-represented regions remains scarce. This imbalance not only limits the generalisation of findings to diverse epidemiological contexts but also undermines the ability of mathematical models to generate policy-relevant insights for populations facing the highest burden of AMR, particularly in the Global South.

The predominance of bacterial pathogens in the reviewed models reflects their central role in the global burden of AMR [5]. Bacteria are the primary targets of antimicrobial use in both human and animal health, and they account for the most pressing clinical challenges associated with drug resistance, including MDR and XDR strains [4, 6]. Major global initiatives, such as the World Health Organization’s (WHO) Global Antimicrobial Resistance and Use Surveillance System (GLASS) [2], the 2024 WHO Bacterial Priority Pathogens List [122], and the United States Centers for Disease Control and Prevention’s (CDC) threat reports [123], prioritise bacterial species like *E. coli*, *S. aureus*, *K. pneumoniae*, and *P. aeruginosa* because of their high prevalence, clinical impact, and resistance profiles, the focus of modelling on these pathogens is therefore aligned with these global priorities. In addition to their clinical and epidemiological relevance, the importance of these pathogens in modelling studies can also be attributed to ecological, experimental, and methodological factors. *E. coli*, for example, is often investigated because it acts as an indicator organism for faecal contamination and antimicrobial resistance surveillance in human and environmental samples [124]. Similarly, *S. aureus* has been prioritised due to its recognition as an important nosocomial pathogen, the existence of well-established experimental models, and the availability of extensive longitudinal data [125]. Furthermore, these pathogens are relatively easy to cultivate in the laboratory, have extensively characterised genomes, and have established methods for detecting resistance genes, which facilitates both experimental parameterisation and empirical validation of models [126–128]. In contrast, viral and fungal AMR have received less modelling attention, despite growing concerns over resistance in viruses such as HIV and influenza [129], and in fungi such as *C. auris*, which the CDC lists as an urgent threat [130]. This highlights an important limitation in current modelling efforts, particularly the lack of frameworks that capture how different antibiotic classes interact with the host microbiota and influence pathogen dynamics, including non-bacterial agents.

The omission of the host immune response (included in only eight studies) and PK/PD modelling (absent in *50%* of studies) represents significant challenges for translating model predictions into clinical practice. Host immunity is central to infection clearance and influences the selection window for resistance. Moreover, the lack of PK/PD frameworks directly limits the capacity of models to assess drug-dosing strategies and optimize clinical outcomes, thereby reducing their usefulness in developing actionable therapeutic guidelines against MDR/XDR strains.

The scarcity of studies that integrate economic dimensions constitutes one of the most critical limitations for policy translation. This omission represents a fundamental barrier, as without cost-effectiveness or macroeconomic analyses, models fail to provide the quantifiable evidence required by policymakers to justify substantial investments in AMR control and intervention programs, particularly in LMICs where the economic burden is relatively high. Given the projected economic losses associated with AMR, including estimated annual reductions of up to *1%* in global GDP and even more severe impacts, between *5%* and *7%*, in developing countries [114, 131], the integration of economic and epidemiological frameworks into future modelling efforts is essential to strengthen their policy relevance. Furthermore, AMR-related effects are expected to extend to strategic sectors such as international trade and animal production, where increasing resistance to antibiotics used in livestock is projected to reduce the global supply of animal-derived food (e.g., meat, milk, and eggs) by approximately *11%* by 2050, with the greatest repercussions in LMICs [114, 132–134].

### Key findings

The findings of this scoping review reveal a consistent pattern in how mathematical modelling has been applied to study AMR over the past six years. The use of mathematical models to study AMR has remained active over the past six years, with a focus on deterministic models, particularly those based on ordinary differential equations (ODEs). Almost all of the included studies focused on bacterial pathogens, especially *P. aeruginosa*, *E. coli*, and *S. aureus*, demonstrating global health priorities, given that bacteria are the main pathogens that cause AMR. In contrast, viral, fungal, and parasitic resistance remains neglected in mathematical modelling studies. Over half of the studies focused on human hosts, whereas animal and environmental contexts were rarely considered. Only one of the studies adopted a One Health perspective, employing a multiscale modelling approach. With respect to the mechanisms of resistance acquisition, mutation was the most frequently modelled pathway, followed by horizontal gene transfer (HGT), especially via conjugation, whereas transformation and transduction were rarely incorporated. Approximately half of the studies did not incorporate pharmacokinetic/pharmacodynamic (PK/PD) modelling. Few studies used PK/PD modelling simultaneously, while others have modelled only one of the components. Critical biological factors, such as host immune response and spatial dispersion, have rarely been explored and are underrepresented in the models, despite their epidemiological relevance. Therapeutic strategies were predominantly modelled as monotherapy, with combination therapies of two or more drugs rarely included in the models. While external validation has commonly attempted, economic impact assessments and future scenario projections have rarely been included in model outputs. A significant geographic imbalance was observed, with most studies conducted by institutions in HICs. Contributions from the Global South remain limited, restricting the generalisability of the results to diverse epidemiological contexts. These important findings show where mathematical modelling of AMR has focused and consolidated in recent years, highlighting its strengths and aspects that need further exploration and guiding priorities for future model development and interdisciplinary collaboration.

A number of persistent methodological presumptions should be taken into consideration when interpreting the conclusions derived from these models. For instance, the broad assumption of homogeneous mixing suggests that every single individual or microbial cell has an equal chance of interacting with each other, which is rarely the case in practical settings. Due to its disregard for the effects of social networks and spatial structure, this can skew estimates of the rate at which resistance propagates. Moreover, the adaptive character of biological and behavioural systems is overlooked when parameters are set to fixed values (e.g., constant transmission or mutation rates). Because these rates are actually dynamic and subject to change in response to interventions, model predictions may be unduly optimistic or pessimistic. Moreover, the limited treatment pathways, mainly focusing on monotherapy, make for a simple clinical picture, whereas the reality is often treatment with two or more medicines. The models may fall short of capturing key dynamics such as collateral sensitivity or resistance suppression that are relevant to combined treatments, sequential treatments, and patient adherence. So, although these models can provide us with theoretical insights, we should only consider their predictions as indicative scenarios under certain conditions that may be simplified. It is important to recognise these hindrances to ensure responsible translation of modelling findings into policy and practice.

### Research gaps

The dominance of deterministic ODEs suggests a serious methodological gap caused by the missing model components that account for stochasticity, rare events, and individual-level heterogeneity, which are critical to real-world AMR emergence and spread dynamics. In addition, the dearth of One-Health models with only one paper that brings together human, animal, and environment compartments represents a conceptual gap which restricts our understanding of cross-sector transmission feedbacks.

Feeding pathway single-focus (e.g., mutation, conjugation) resistance acquisition might overlook synergies multiple-concurrent mechanisms such as transduction and transformation. As our results show, the limited application of PK/PD frameworks indicates a missed opportunity to link drug concentrations with resistance development. A key constraint to policy relevance is the lack of economic impact assessments of AMR and spatial heterogeneity in models. The latter neglects the financial burden of AMR and geography’s role in resistance formation. To go beyond description to predictive and policy-relevant models, it is important to fill the gaps.

### Public health implications and policy relevance

The results of this scoping review have direct implications for public health practice and policy development. First, the extensive use of deterministic models limits the ability to address complex local outbreaks. The lack of stochastic and spatially explicit models weakens prediction and outbreak control, as hospital teams need to know not only the average number of cases but also the probability of an outbreak in a specific sector. Decisions on isolation and resource allocation are often made without a quantitative framework that accounts for rare events and transmission across interconnected units.

Second, most models neglect the One Health perspective, ignoring environmental and animal reservoirs. This limitation undermines AMR action plans, as policies restricting antibiotic use solely in human health may be ineffective. Future initiatives should adopt multisectoral models that replicate resistance transmission across human, animal, and environmental systems.

Third, few studies consider the economic impact of AMR. Without cost-benefit analyses or economic projections (e.g., reduced hospitalisations, productivity gains), models fail to guide resource allocation and funding decisions. Combining epidemiological and economic perspectives is necessary to translate science into policy action.

Finally, research is geographically imbalanced, most models come from high-income countries, limiting applicability to LMICs where AMR burden is often higher. Public health leaders in the Global South need locally calibrated tools, not adapted models from North America or Europe. Enhancing research capacity and data-sharing systems in these regions is a policy priority. In conclusion, future research must move beyond theoretical exercises to become decision-support tools that incorporate ecological and economic dimensions, address structural issues of cost and inequality, and support global AMR control efforts.

### Conclusion

By mapping recent literature on the mathematical modelling of AMR, this scoping review revealed a developing field, but one still has significant methodological and conceptual discrepancies. We found a predominance of deterministic models based on ODEs, which, although useful for initial analysis, reveal a disconnect from the stochastic and heterogeneous nature of the evolution and spread of AMR. Similarly, the almost exclusive focus on bacterial pathogens and the mechanisms of mutation and HGT–conjugation overlooks essential components of the problem, such as AMR in viruses and fungi and the contribution of other gene transfer pathways.

Our findings show an under-representation of One Health models. The near absence of models that integrate human, animal, and environmental components prevents a comprehensive understanding of AMR transmission cycles. For mathematical models to become truly effective decision support tools, future research needs to incorporate the One Health approach. The inclusion of animal and environmental reservoirs in the models would enable the creation of integrated action plans, preventing antibiotic restriction policies from being limited to human health alone, thereby increasing the overall effectiveness of AMR mitigation strategies. Furthermore, the rare incorporation of economic impact modelling in analyses limits the usefulness of models for policymakers who need to quantify the costs associated with AMR.

Therefore, we conclude that advances in mathematical modelling applied to AMR require conceptual and methodological shifts. Models must be able to capture the complexities associated with AMR, requiring the incorporation of stochastic and hybrid approaches, expanding their application to non-bacterial pathogens, and integrating the One Health perspective and socioeconomic variables. This conceptual shift is crucial for models to fully realise their potential as predictive tools and provide policymakers with the evidence needed to formulate more effective, realistic, and globally equitable intervention strategies.

### Limitations and future directions

This review is limited to articles published in English, which may exclude valuable contributions in other languages. Furthermore, although this scoping review focused on mapping trends and identifying gaps in the mathematical modelling of AMR, it did not include a critical assessment of methodological quality. Additionally, no formal protocol was registered a priori in databases such as OSF, which may reduce transparency and reproducibility, despite adherence to an internal protocol. Moreover, only publications indexed in three databases (PubMed, Web of Science, and Scopus) were considered, and the specific Boolean search string used may have influenced the retrieval of studies, possibly omitting relevant works. The review period *(2019-2024)* was selected to capture recent advances but may have excluded seminal older studies that remain methodologically relevant.

Moreover, this review emphasises dynamic mechanistic models (e.g., differential equation or agent-based formalism type) to understand the processes driving AMR. As a result, we did not include studies that employed purely statistical models (e.g. GLMs or GAMs), time-series analyses (e.g., ARIMA/SARIMA), or artificial intelligence/machine learning algorithms. While these data-driven approaches are important for pattern, prediction, and correlation identification in large datasets, they are not mechanistic models. We focused on synthesising research representing the causal mechanisms, such as mutation, horizontal gene transfer, and selection, that drive AMR dynamics. Future scoping or systematic reviews could be undertaken, specifically directed at these data-driven approaches, which would complement the current body of modelling work in AMR research.

Another important limitation revealed by this review concerns the scarcity of mathematical representation of therapeutic strategies involving antibiotic combinations with distinct mechanisms of action. Although combination therapy and synergistic or antagonistic interactions are widely used in clinical practice to delay resistance, most models assume independent drug effects or restrict analyses to monotherapies. The incorporation of multi-drug interactions, including suppressive or competition-mediated effects, remains an open line of research with significant potential to improve predictions of resistance evolution. An additional aspect that none of the reviewed studies considered was the ecological impact of antimicrobial treatment on the host-associated microbiota. Antibiotic-induced dysbiosis can weaken colonization resistance, alter competitive dynamics, and indirectly promote the expansion or acquisition of resistant strains [135, 136]. Within the 36 studies published between 2019 and 2024 included in this review, none explicitly incorporated ecological interactions at the microbiota level or antibiotic-induced dysbiosis affecting competitive dynamics. This absence highlights a potential gap in recent mechanistic AMR modelling trends and indicates an area for further development to better capture the emergence and persistence of resistance.

Future work should explore a broader inclusion of AMR modelling studies beyond the English-language literature and conduct comparative analyses between different modelling approaches (e.g., deterministic vs. stochastic models); develop more complex and realistic models that incorporate heterogeneous populations, multiple resistance genes, and treatments considering more than one type of drug; increase the use of stochastic, hybrid, or individual-based models to reflect real-world dynamics; incorporate components into the models that seek to assess the real economic impact that AMR has on a country; and encourage the development of models that integrate the human, animal, and environmental sectors in accordance with the One Health framework. On the basis of the insights presented here, we also intend to develop a mathematical model of AMR in future work, aiming to address some of the gaps highlighted by this review.

## Supplementary Information

Supplementary Material 1.

## Data Availability

The datasets generated and/or analysed during the current study are available in the GitHub repository: amr_modelling_scoping_review.

## Abbreviations

ABMs: Agent-based models
ADME: Absorption, distribution, metabolism, excretion
AMR: Antimicrobial resistance
CDC: Centers for disease control and prevention
GDP: Gross domestic product
GLASS: Global Antimicrobial Resistance and Use Surveillance System
HGT: Horizontal gene transfer
HICs: High-income countries
HIV: Human immunodeficiency virus
ICU: Intensive care unit
IDEs: Integro-differential equations
JBI: Joanna Briggs Institute
LMICs: Low- and middle-income countries
LNA: Linear noise approximation
MDR: Multidrug resistance
MIC: Minimum inhibitory concentration
N/A: Not applicable
OECD: Organization for Economic Cooperation and Development
ODEs: Ordinary differential equations
OSF: Open science framework
PCC: Population, concept, context
PDEs: Partial differential equations
PK/PD: Pharmacokinetic/Pharmacodynamic
PRISMA-ScR: Preferred reporting items for systematic reviews and meta-analyses extension for scoping reviews
R[NONSPACE: &D] Research and development
SDEs: Stochastic differential equations
WHO: World health organization
XDR: Extensively drug-resistant
*A. baumannii*: *Acinetobacter baumannii*
*C. difficile*: *Clostridioides difficile*
*E. coli*: *Escherichia coli*
*K. pneumoniae*: *Klebsiella pneumoniae*
*P. aeruginosa*: *Pseudomonas aeruginosa*
*S. aureus*: *Staphylococcus aureus*
*S. pneumoniae*: *Streptococcus pneumoniae*
