# Supplementary material for: Mapping the landscape of mathematical models for antimicrobial resistance: a scoping review
Source: BMC Public Health. 2026 Jul 2;26:2835. doi: 10.1186/s12889-026-27533-8 (PMC13643989; doi:10.1186/s12889-026-27533-8)
Supplement: Supplementary file 1 [file 12889_2026_27533_MOESM1_ESM.pdf]

Supplementary Material

Appendix A Evaluation aspects of each study 36
Appendix B Code and data availability 36

Appendix A Evaluation Aspects of Each Study

To analyse the studies included in this scoping review, we developed a set of structured questions organized into three main categories: (i) Model Type and Context, (ii) Model Construction and Correlated Parameters, and (iii) Model Outputs and Validation. The purpose of these questions is to guide the extraction of relevant information about mathematical models of AMR, allowing consistent comparisons across studies. Each question was formulated to allow objective and comparable responses, ensuring transparency and consistency in the characterization of the evaluated models.

MODEL: TYPE AND CONTEXT

1. What is the nature of the model used in the study (deterministic, stochastic, agent-based, etc.)?
2. What is the main mathematical structure of the model (e.g., ODEs, PDEs, etc.)?
3. Which host species are considered (e.g., humans, animals, etc.)?
4. At what spatial or contextual scale is the model applied (e.g., individual, hospital, community, etc)?
5. Which classes of pathogens are modelled (e.g., bacteria, viruses, fungi, parasites)?
6. What is the specific pathogen considered in the model, when applicable (e.g., *Mycobacterium tuberculosis*, *Staphylococcus aureus*)?

MODEL: CONSTRUCTION AND CORRELATED PARAMETERS

1. Does the model include explicit spatial components (e.g., contact networks, dispersal between regions)?
2. Does the model incorporate fitness costs (adaptive advantages/disadvantages) for resistant strains?
3. Does the model include host immune response dynamics?
4. Which mechanisms of resistance acquisition are considered (e.g., mutation, horizontal gene transfer - HGT, etc.)?
5. Does the model address resistance to multiple antimicrobials simultaneously (multidrug resistance)?
6. Does the model incorporate pharmacokinetic/pharmacodynamic (PK/PD) parameters to describe antimicrobial action?

MODEL: OUTPUTS AND VALIDATION

1. Was a sensitivity analysis performed to assess the influence of model parameters?
2. Are model results validated with empirical data or compared to existing models?
3. Does the model capture the temporal dynamics of resistance under prolonged antimicrobial exposure?
4. Does the model use antimicrobial efficacy parameters (e.g., Minimum Inhibitory Concentration - MIC)?
5. Does the model evaluate the cost-effectiveness or economic impacts of the analysed interventions?
6. Does the model consider monotherapy, combination therapy, or both?
7. Can the model results be used in practical applications (e.g., scenario forecasting, decision support, intervention planning)?

Appendix B Code and data availability

The code and data used in this study are available in the GitHub repository: amr_modelling_scoping_review.
